# Disseminated cryptococcosis in a deceased with HIV‐1 diagnosed by minimally invasive tissue sampling technique

**DOI:** 10.1002/ccr3.3865

**Published:** 2021-02-02

**Authors:** Nuwadatta Subedi, Suraj Bhattarai, Sunita Ranabhat, Binita K. Sharma, Madan P. Baral, Tirtha L. Upadhyaya

**Affiliations:** ^1^ Department of Forensic Medicine Gandaki Medical College Pokhara Nepal; ^2^ DECODE‐MAUN Research Project Gandaki Medical College Pokhara Nepal; ^3^ Department of Pathology Gandaki Medical College Pokhara Nepal; ^4^ Department of Microbiology Gandaki Medical College Pokhara Nepal; ^5^ Department of Forensic Medicine Pokhara Academy of Health Sciences Pokhara Nepal; ^6^ Department of Internal Medicine Gandaki Medical College Pokhara Nepal

**Keywords:** disseminated cryptococcosis, HIV/AIDS, minimal invasive tissue sampling, Nepal

## Abstract

Minimal invasive tissue sampling (MITS) technique detected HIV infection and disseminated cryptococcosis in an adult female with sudden death. A proper autopsy is essential to diagnose the exact cause of death and MITS can suffice in natural deaths.

## Graphical abstract

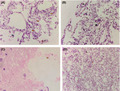

## 1 INTRODUCTION

Cryptococcosis is one of the common mycological conditions leading to morbidity and mortality in patients with AIDS. We are presenting a case of disseminated cryptococcosis in a patient infected with human immunodeficiency virus (HIV‐1), which were both diagnosed postmortem using minimally invasive tissue sampling method.

Cryptococcosis is one of the common mycological conditions leading to morbidity and mortality in patients with AIDS.^1^ Cryptococcus can cause life‐threatening infections like meningoencephalitis and disseminated cryptococcosis in immunocompromised patients.^2^ According to the studies, 10%‐25% of AIDS patients with cryptococcosis die even if they have received antifungal therapy, and 30%‐60% die within the first year of onset of the infection.^1^

We present a case of disseminated cryptococcosis in a patient with HIV, which were both diagnosed by using the Minimally invasive tissue sampling (MITS) technique.^3^ In Nepal, clinical autopsies are not usually conducted and forensic autopsies are not routinely backed up with histopathological, microbiological, and other ancillary tests. In this case, we performed the MITS procedure along with histopathological, serological, and microbiological tests as a part of a research project—Determining Efficiently the Cause of Death among Adults and Generating Mortality Evidence at MITS Alliance Unit Nepal (DECODE MAUN Nepal).

## 2 CASE DETAILS

A 35‐year‐old female patient was allegedly having myalgia for 5 days and was taking analgesics as self‐medication. She was found dead at her own home after a few days of the onset of her symptoms. As the case was a sudden death, it was reported to the law enforcement agencies, and after the necessary inquest, the deceased was brought to our study site for medico‐legal autopsy. According to her relatives, the deceased was married, housewife, living with her two children, and was not reported to be having any underlying systemic diseases or conditions during her lifetime. There was a denial of any significant trauma during her life. She was a nonsmoker, nonuser of smokeless tobacco, and nonalcoholic.

On external examination, there were ill‐defined contusions on the subject’s periorbital region, 5 × 3 cm contusions on both the knees and diffuse 6 × 7 cm contusion on the right thigh. These contusions could be attributed to her staggering efforts to move around in the last few days of her life. There was clotted blood in the perineal and vaginal region, but no injuries were evident; it was consistent with menstrual bleed confirmed by the statements from her family members. On complete autopsy examination, no obvious internal injuries were detected. Both the lungs were mildly edematous with patches of consolidation. The spleen was grossly enlarged to approximately one kilogram in weight and 30 × 25 cm in size. The parenchyma of the spleen was heavily congested.

We collected 20 mL of blood from the subject’s subclavian vein and 10 mL of cerebrospinal fluid (CSF) from the occipital puncture. We collected tissue samples using the MITS technique from the subject’s key organs: brain tissue sample through occipital and transnasal approach, right and left lung tissue, and liver tissue samples. Additionally, we collected spleen tissue as it was grossly enlarged and rectal swab. The first samples from both the right and left lungs were collected using a new sterile needle and placed into Brain Heart Infusion (BHI) broth for culture, then incubated for 72 hours. The subsequent lung and other tissue samples were placed into labeled tissue cassettes, which were subsequently placed into a tissue jar containing 10% neutral buffered formalin solution and then sent for histopathological examination. Initially, the tissue sections were reviewed by hematoxylin and eosin (H&E) stains and later analyzed by Periodic Acid‐Schiff (PAS).

About 3 mL sample of blood was kept into BHI broth and incubated for 7 days. Microscopic examination of CSF sample by Gram’s stain and India Ink preparations was performed. CSF samples were cultured in Blood agar (BA) and Chocolate agar (CA) plates and incubated at 35°C for 7 days. CSF samples were incubated in two tubes of Sabouraud dextrose agar (SDA), and another tube at room temperature. Urea hydrolysis test was performed from the colonies obtained from BA, CA, and SDA.

## 3 RESULTS

**Figure 1:**
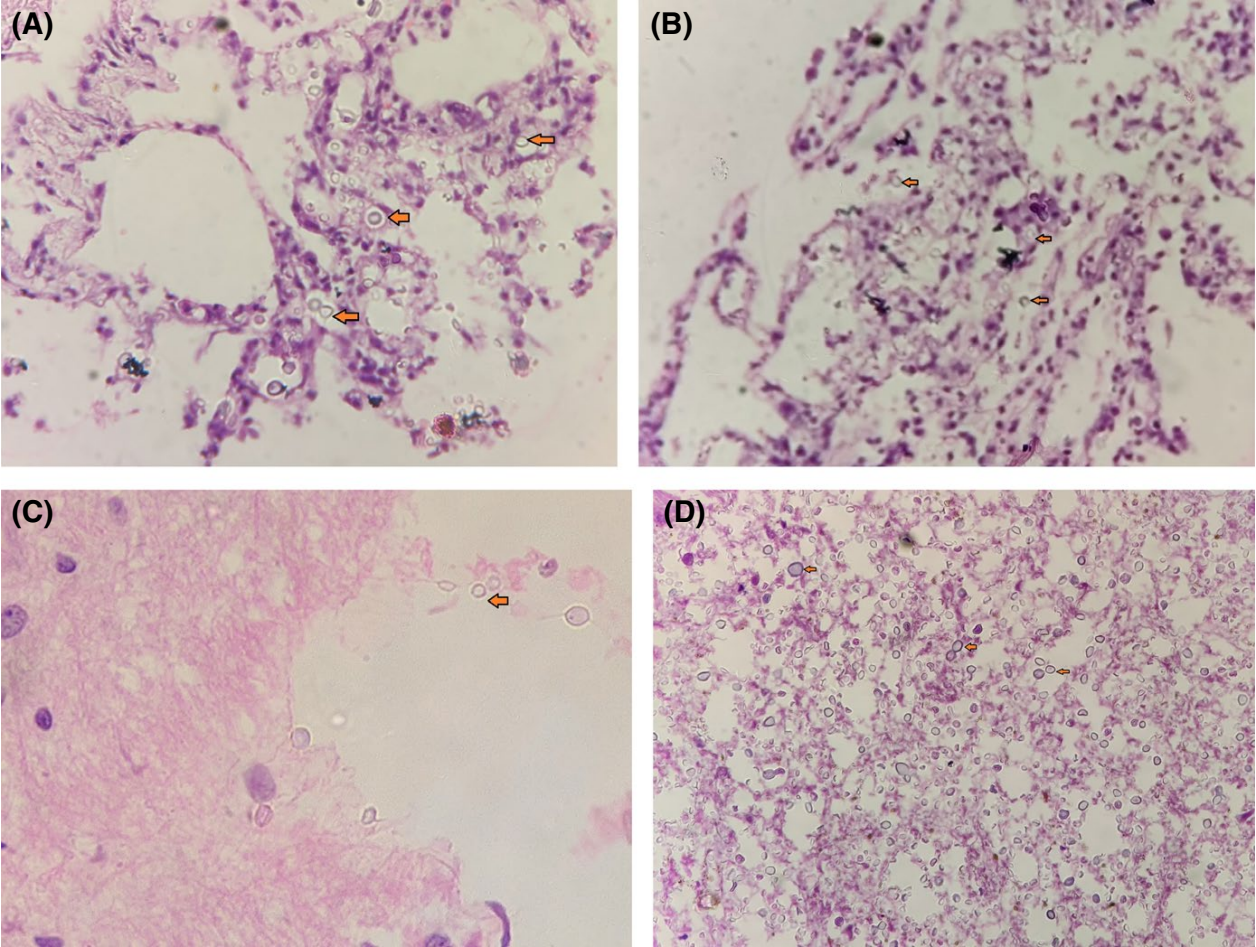
Histopathological findings of tissue specimens collected by MITS technique. A, Right Lung with cryptococci in alveolar spaces (H&E 40×); B, Left Lung with cryptococci in alveolar spaces (H&E 40×); C, Brain tissue with cryptococci (PAS Stain 40×); D, Spleen with dense infiltration with cryptococci (PAS stain 10×)

Histopathological findings (Figure 1): Alveolar spaces of both the right and left lungs were filled with round refractile structures with thick capsule suggestive of cryptococci. There were also a few alveolar macrophages (Figure 1A,B). There were similar structures suggestive of cryptococci in brain tissues too (Figure 1C). The liver was unremarkable, whereas the spleen had areas of extensive congestion along with dense infiltration by cryptococci (Figure 1D).

**Figure 2:**
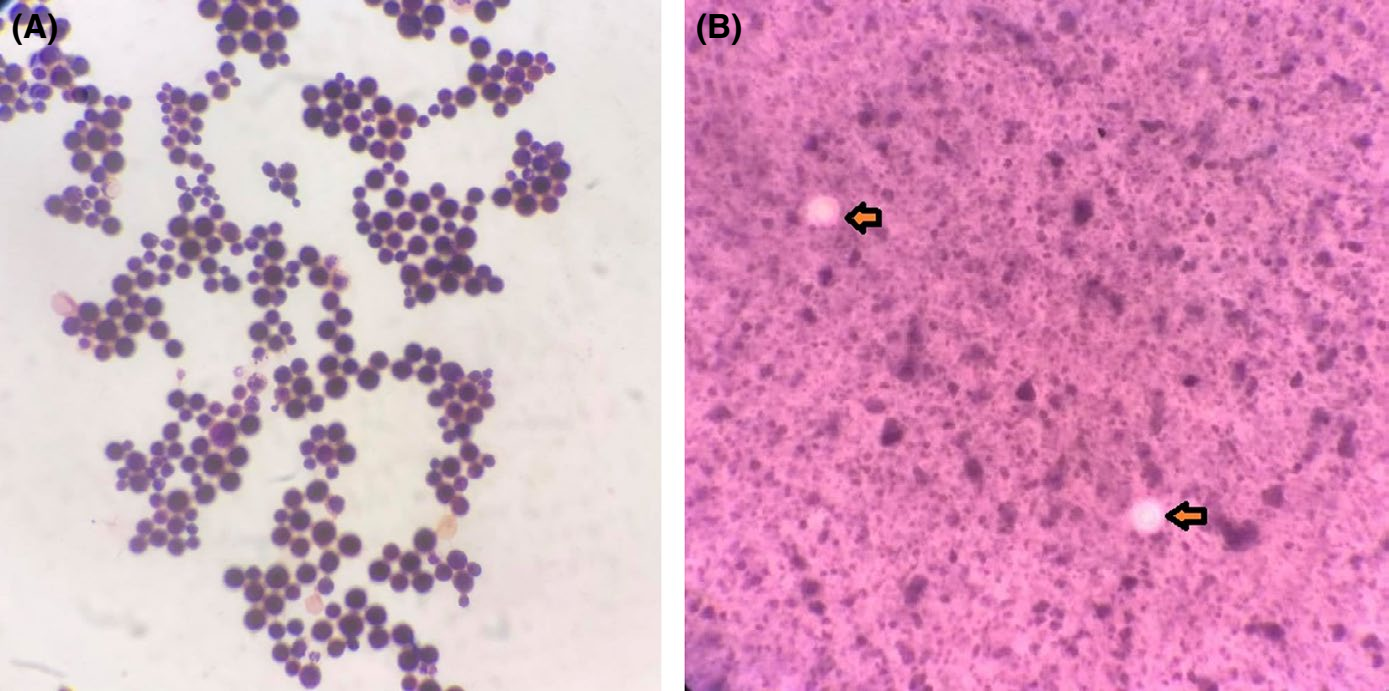
Microbiological findings of CSF specimen. A, Gram’s stain showing Gram‐positive budding round yeast cells suggestive of *Cryptococcus* spp; B, Indian ink staining showing capsule of *Cryptococcus* spp and the zone of clearance or “halo” surrounding the yeast cells

A Peripheral blood smear was negative for malarial parasites. Serology was positive for HIV‐1 antibodies by HIV‐Tri Dot assay, but the serological tests for dengue, malaria, HBsAg, HCV, Brucella, and Leptospira were all negative. Microbiological findings (Figure 2): Gram stain of CSF sample revealed Gram‐positive round yeast cells suggestive of *Cryptococcus* spp (Figure 2A). Typical round encapsulated yeast forms suggestive of *Cryptococcus* spp were demonstrated by India ink preparation of CSF (Figure 2B). Colonies in Sabouraud dextrose agar were creamy, white, and mucoid. Similarly, colonies on blood agar, chocolate agar, and Sabouraud dextrose agar showed Gram‐positive round budding yeast cells suggestive of *Cryptococcus* spp. The Urease test was also positive after 48 hours of incubation. The most remarkable finding was *Cryptococcus neoformans* complex organism detected by CSF culture. Additionally, the lung tissue culture showed the growth of *Proteus miribilis*.

The cause of death was determined according to the World Health Organization International Classification of Diseases (ICD 10) protocol and based on the consensus of “the Cause of Death” panel within our research project. The immediate cause of death in this case (Part IA) was disseminated cryptococcosis (ICD code B45.7) with underlying HIV/AIDS—ICD B20.5 (Part IB). Pneumonia due to *Proteus mirabilis* (J15.6) was the secondary condition (Part II) that was not directly related to death.

## 4 DISCUSSION

We have presented a case of disseminated cryptococcosis in an HIV‐infected woman of 35 years of age. Neither HIV nor cryptococcosis had been diagnosed during her lifetime. Therefore, our finding highlights the importance of performing proper laboratory and pathology investigations at the time of autopsy to determine the cause of death. In Nepal and in many low‐ and middle‐income countries, clinical autopsies are usually not conducted, so the cause of death remains undiagnosed in many cases. Forensic autopsies are conducted only when there is an official request from the Law enforcement agencies, mostly in unnatural and sudden deaths. Even forensic autopsies are not routinely backed up with ancillary investigations which lead to the undetermined causes of deaths. In such cases, histopathological, microbiological, and other relevant tests in human specimens could have contributed to establish the cause of death. From microbiological perspective, this gap in evidence indicates the need for taking proper precautions against potentially contagious infections like HIV during an autopsy and regards every case as potentially infectious unless proven otherwise. The diagnosis of HIV in the married female with two children can have significant implications for the family members, as the husband could have also contracted the disease and there could have been vertical transmission to her children too. In this context, our team has counseled the family members to perform HIV test and seek treatment if needed according to the protocol. The condition of disseminated cryptococcosis implies that the deceased could be in the immunocompromised state for a long time.

﻿The periorbital contusions and the contusions on the thighs in the absence of any internal injuries rule out significant trauma. Gross examination during the autopsy failed to attribute the cause of death, but the ancillary investigations were useful to establish disseminated cryptococcosis as the final or direct cause of death with HIV/AIDS as an underlying cause. Cryptococcus causes meningoencephalitis and disseminated cryptococcosis usually in immunocompromised hosts,^4^, ^5^ though cases of cryptococcal meningitis have also been reported in immunocompetent patients.^6^, ^7^, ^8^ The research on the prevalence of cryptococcosis is very sparse in literature from Nepal. In a record review of one fiscal year in the major tertiary center of Nepal, among 15 patients with Cryptococcal meningoencephalitis, the majority (9, 60%) had HIV infection.^5^

The involvement of cryptococcus in causing death among HIV‐infected patients was demonstrated in approximately 10% of cases by an autopsy study in low‐income settings in Mozambique.^9^ The pathology of cryptococcal meningoencephalitis with minimal inflammatory infiltrates and gelatinous pseudocysts produced by abundant *C*. *neoformans* was shown in most of the cases in an autopsy study in HIV‐infected patients.^10^

There are several ways of diagnosing cryptococcosis. CSF culture is regarded as the gold‐standard method and microscopy can be added to aid in the diagnosis. In recent years, there has been an evolution of cryptococcal antigen (CrAg) testing which has proven to be a relatively inexpensive and more sensitive method. To facilitate easy visualization of the fungus in the specimens, stains like India ink are also used.^11^ The India ink staining for fungus detected cryptococcus in our case as well. The sensitivity of India ink staining to detect cryptococcus is up to 80% in HIV‐positive patients.^12^

Postmortem examination should be done meticulously, and the use of ancillary tests should be made available to attribute mortality to specific pathogens. The accurate diagnosis of the cause of death is important as it informs the burden of disease in the region and supports the surveillance system of the country. Moreover, it can make a great impact on public health policies and helps in the prophylaxis and treatment modalities, particularly in infectious diseases.

## 5 CONCLUSION

Disseminated cryptococcosis was attributed to the final cause of death in the deceased with underlying HIV disease, who was not diagnosed with either of these conditions during her lifetime. Autopsy should be conducted meticulously along with adequate ancillary tests to correctly identify the cause of death as it can provide valuable information about disease burden in the region. MITS technique can be a useful tool in determining the cause of death in low resource settings. In mortality cases with likely infectious conditions and other natural diseases, clinical autopsies have important clinical and public health implications.

## CONFLICT OF INTEREST

This is one of the cases as a part of a research project—Determining Efficiently the Cause of Death among Adults and Generating Mortality Evidence at MITS Alliance Unit Nepal (DECODE MAUN Nepal) supported by MITS Surveillance Alliance.

## AUTHOR CONTRIBUTIONS

NS: performed MITS, participated in cause of death determination, and prepared the first draft of the manuscript. SB: reviewed clinical information of the case, participated in cause of death determination, and contributed to manuscript writing. SR: reported pathological tests and participated in cause of death determination and reviewed the draft of the manuscript. BK: reported microbiological tests and participated in cause of death determination and reviewed the draft of the manuscript. MPB: performed MITS, participated in cause of death determination, and reviewed the draft of the manuscript. TLU: reviewed clinical information of the case, participated in case discussion for the cause of death determination, and reviewed the draft of the manuscript.

## INFORMED CONSENT

Written informed consent was taken from the nearest family member of the deceased for publishing the case details and associated laboratory images.

## Data Availability

Data sharing does not apply to this article as no datasets were generated or analyzed from this single case.

## References

[1] Jarvis JN , Harrison TS . HIV‐associated cryptococcal meningitis. AIDS. 2007;21(16):2119‐2129. 10.1097/QAD.0b013e3282a4a64d 18090038

[2] Maziarz EK , Perfect JR . Cryptococcosis. Infect Dis Clin North Am. 2016;30(1):179‐206. 10.1016/j.idc.2015.10.006 26897067 PMC5808417

[3] What Is MITS ‐ MITSPortal. https://mitsalliance.org/WhatIsMITS. Accessed November 8, 2020.

[4] Aldhaleei WA , Bhagavathula AS , Alhajeri A . Vision loss in an AIDS patient with cryptococcal meningitis. Med Mycol Case Rep. 2019;24(April):51‐53. 10.1016/j.mmcr.2019.04.001 31032178 PMC6479074

[5] Kharel G , Ojha R . Spectrum of cryptococcal meningoencephalitis in tertiary hospital in Nepal spectrum of cryptococcal meningoencephalitis in tertiary hospital in Nepal. J Inst Med. 2018;40(2):27‐32.

[6] Jha A , Adhikari S , Sigdel KR , et al. Case report: cryptococcal meningitis in an apparently immunocompetent patient in Nepal‐challenges in diagnosis and treatment. Wellcome Open Res. 2019;4:1‐16. 10.12688/wellcomeopenres.15187.2 31289752 PMC6600853

[7] Khanal B , Sharma SK , Deb M . Cryptococcal meningitis in a non‐AIDS patient. J Nepal Med Assoc. 2003;41(142):323‐325. 10.31729/jnma.759

[8] Radheshyam KC , Karkey A , Prajapati KG , Baker S , Basnyat B . Fatal cryptococcal meningitis in a HIV‐seronegative patient with liver cirrhosis. JMM Case Reports. 2014;1(3):10‐12. 10.1099/jmmcr.0.001982

[9] Hurtado JC , Castillo P , Fernandes F , et al. Mortality due to Cryptococcus neoformans and Cryptococcus gattii in low‐income settings: an autopsy study. Sci Rep. 2019;9(1):1‐10. 10.1038/s41598-019-43941-w 31097746 PMC6522501

[10] Klock C , Cerski M , Goldani LZ . Histopathological aspects of neurocryptococcosis in HIV‐infected patients: autopsy report of 45 patients. Int J Surg Pathol. 2009;17(6):444‐448. 10.1177/1066896908320550 18611927

[11] Nalintya E , Kiggundu R , Meya D , Road MH . Evolution of cryptococcal antigen testing. What is new? Curr Fungal Infect Rep 2017;10(2):62‐67. 10.1007/s12281-016-0256-3. PMC4858186 27158322

[12] O’Halloran JA , Powderly WG , Spec A . Cryptococcosis today: it is not all about HIV infection. Curr Clin Microbiol Rep. 2017;4(2):88‐95. 10.1007/s40588-017-0064-8 29130027 PMC5677188

